# The Effects of Systemic Tranexamic Acid Administration on Drainage Volume, Length of Hospital Stay, and Postoperative Complications in Reduction Mammaplasty

**DOI:** 10.3390/jcm14010151

**Published:** 2024-12-30

**Authors:** Sara Magni, Leon Guggenheim, Geraldine Fournier, Corrado Parodi, Alberto Pagnamenta, Daniel Schmauss, Yves Harder

**Affiliations:** 1 Department of Plastic, Reconstructive and Aesthetic Surgery, Ospedale Regionale di Lugano, Ente Ospedaliero Cantonale (EOC), 6900 Lugano, Switzerland; saramagnimd@gmail.com (S.M.); corrado.parodi@eoc.ch (C.P.); daniel.schmauss@eoc.ch (D.S.); 2 Faculty of Biomedical Sciences, Università della Svizzera Italiana, 6900 Lugano, Switzerland; leon.guggenheim@eoc.ch (L.G.); alberto.pagnamenta@eoc.ch (A.P.); 3 Department of General Surgery, Spital Maennedorf, 8708 Maennedorf, Switzerland; geraldine.fournier@outlook.com; 4 Clinical Trial Unit (CTU), Ente Ospedaliero Cantonale (EOC), 6900 Lugano, Switzerland; 5 Department of Plastic, Reconstructive and Aesthetic Surgery and Hand Surgery, Centre Hospitalier Universitaire Vaudois (CHUV), 1011 Lausanne, Switzerland; 6 Faculty of Biology and Medicine, University of Lausanne (UNIL), 1015 Lausanne, Switzerland

**Keywords:** reduction mammaplasty, breast reduction, tranexamic acid, drainage, postoperative complications

## Abstract

**Background:** Reduction mammaplasty is a common, elective, and safe operation, usually executed in healthy patients. Nonetheless, postoperative complications like bleeding and seroma formation can occur and significantly complicate the postoperative course. Tranexamic acid (TXA), a commonly used antifibrinolytic drug, offers a novel approach to reduce these complications. This study aims to evaluate its effect on the rate of postoperative bleeding, drainage volume, length of hospital stay, and other postoperative complications in patients undergoing reduction mammaplasty. **Method:** A retrospective study on all patients undergoing reduction mammaplasty at the Department of Plastic, Reconstructive, and Aesthetic Surgery EOC between 2015 and 2022 was conducted. Patients were divided into the TXA group receiving systemic TXA for 48 h and the control group not receiving any TXA. All data were analyzed using nonparametric formulas. **Results:** A total of 209 breasts were included in the study, with 138 cases in the control group and 71 in the TXA group. Three cases requiring revision surgery due to bleeding were observed in the control group, whereas none were observed in the TXA group. Total drainage volume was significantly reduced in the TXA group compared to the control group (TXA: 41.6 mL vs. control: 53.8 mL; *p* = 0.012), resulting in a significant reduction in length of hospital stay (TXA: 1.6 days vs. control: 2.2 days; *p* = 0.0001). **Conclusions:** TXA is a well-tolerated drug that significantly reduces postoperative bleeding and drainage volume, resulting in earlier drain removal and reduced length of hospital stay. TXA should, therefore, be widely used in plastic surgery, especially as trends in healthcare systems necessitate more outpatient procedures and quicker postoperative recovery.

## 1. Introduction

Reduction mammaplasty, a highly effective and safe surgery usually performed on otherwise healthy patients, is the procedure of choice to treat macromastia, a condition characterized by hypertrophy of the glandular tissue in the breast. Macromastia poses a significant burden to the affected patients due to the constant physiological and psychosocial distress it can cause [1,2,3,4,5]. Despite the development of numerous techniques designed to reduce breast size while preserving the shape, sensation, and lactation function of the breast, postoperative complications are still a frequent problem. They include, among others, bleeding requiring revision surgery and seroma formation with an incidence of approximately 1.0–9.3% and 1.0–3.0%, respectively [6,7,8].

While seroma formation and dehiscence not requiring surgical revision are considered minor complications after reduction mammaplasty, they may evolve into major complications such as deep infections, bleeding, and necrosis of the nipple–areolar-complex (NAC) requiring surgical revision [9], resulting in a longer postoperative recovery time and eventually impaired aesthetic result [10]. Given the elective nature of the procedure and the potential risk of complications, it remains crucial to identify risk factors and develop new pharmacological or surgical strategies to reduce these adverse events.

The current literature, however, offers only limited evidence for risk factors and prevention of bleeding requiring revision surgery in reduction mammaplasty. While poor control of postoperative hypertensive spikes is an established risk factor with a statistically significant correlation to hematoma formation, the role of intraoperative hypotension has recently become controversial [6,11]. Furthermore, Stanek et al. also challenged the long-held belief on the role of ketorolac, a cyclooxygenase 1 and 2 (COX1 and COX2) blocker known under the name of Toradol, demonstrating its role as an anti-aggregating drug when administered perioperatively mainly to treat pain [11]. However, a consensus is yet to be found on the association between age and hematoma rate due to different results published over the years [6,11,12]. Other factors known to be associated with the most common complications for reduction mammaplasty, like body mass index (BMI), diabetes, previous radiotherapy, smoking, resection weight, and surgical technique, have not been shown to be statistically related to the development of bleeding requiring revision surgery [11,12]. More specifically, the placement of postoperative drains was also analyzed as a risk factor for bleeding but has not been shown to play a statistically significant role in predicting the development of hematomas [6].

Additionally, regarding risk factors for seroma, the current literature is rather scarce. Since there is no clear definition of seroma, results across different studies may vary and, at times, contradict each other. However, current scientific evidence suggests BMI, age over 50 years, amount of tissue resected, secondary surgery, and type of reduction mammaplasty as potential risk factors for increased drainage output and seroma formation [13,14,15,16].

Tranexamic acid (TXA), a common antifibrinolytic drug, poses a potential novel approach to reducing postoperative complications. Multiple studies have already established the ability of TXA to significantly reduce blood loss in different clinical settings, from major trauma to post-partum bleeding and menorrhagia, as well as orthopedic and cardiac surgery [17,18,19,20,21,22]. However, its effect on drainage volume and seroma incidence has not been investigated as extensively and is becoming increasingly important, especially given the increasing use of TXA in plastic surgery.

TXA is a synthetic analog of the amino acid lysine and exhibits both antifibrinolytic and anti-inflammatory properties. The antifibrinolytic effect relies on its ability to hinder the activation of plasminogen into plasmin by blocking its lysine-binding site, thus inhibiting the proteolytic degradation of fibrin clots [23]. Additionally, it is also a direct inhibitor of plasmin [24]. The anti-inflammatory effect of TXA is believed to work through multiple mechanisms, among others, by inhibiting the proteolytic effect of plasmin, which degrades fibrin and causes the generation of pro-inflammatory fibrin fragments [25]. Moreover, TXA has been shown to reduce several pro-inflammatory cytokines including IL-10 [26], IL-6 [27,28,29], and TNFα [27,28,30]. Thus, by suppressing these pathways and inhibiting their peripheral effects, TXA is believed to reduce drainage and postoperative complications.

Although TXA is a commonly used drug, its administration in plastic surgery still presents an off-label use. Thus, many surgeons face the concern of increasing thromboembolic risk. Previous studies have shown, however, that TXA does not increase the risk of thromboembolic events if administered in dosages comparable to that used in this study and respecting the given contraindications, i.e., preexisting allergy to TXA, previous thromboembolic events, and active thromboembolic disease [31,32].

Since many benefits have been experienced using TXA for other indications in surgery, a specific TXA regimen has been implemented in our department since July 2020 for patients undergoing reduction mammaplasty consisting of a specific protocol lasting for 48 h aimed at reducing the incidence of postoperative bleeding requiring revision surgery and seroma-formation.

As the initial observation in patients undergoing reduction mammaplasty and receiving TXA systemically was promising and the scientific evidence was scarce at that time, a retrospective analysis was conducted to assess the exact effects of TXA in these patients.

The aim of this study is to evaluate the effects of TXA on the incidence of bleeding requiring revision surgery in patients undergoing reduction mammaplasty. The secondary outcomes were the total drainage volume, the time until drain removal, the rate of seroma formation, and wound dehiscence.

## 2. Materials and Methods

A mono-centric retrospective study has been conducted in our department, including 157 patients undergoing unilateral or bilateral reduction mammaplasty between October 2015 and October 2021. This study was conducted according to the STROBE guidelines and in accordance with the Declaration of Helsinki. All patients signed a written consent to participate in the study.

This study was approved by the local ethics committee with the BASEC number 2021-01918.

Any consenting patient above the age of 16 undergoing primary uni- or bilateral reduction mammaplasty in the mentioned time period was included in the study. No age limit was set. The age range varied between 16 and 87 years old.

The exclusion criteria were pregnancy and known coagulopathies. Patients who presented with contraindications for TXA, such as hypersensitivity reactions to TXA in the past, positive medical history for seizures, or past thromboembolic events, were included in the control group.

In oncologic patients undergoing bilateral surgery, only the unaffected side was included to avoid the influence of potential lymph node surgery on the total drainage volume.

Patients were divided into TXA and control group. The case group included 71 breasts that were operated on after July 2020 and received TXA as per the mentioned change in regimen according to the following protocol: 1 g i.v./8 h for the first 24 h and 1 g p.o./8 h for the next 24 h. The first dose was administered intraoperatively by the anesthesiologist at the beginning of the operation. After a total of 48 h, drug administration was suspended. The control group included 138 breasts that were operated on mainly before July 2020 and did not receive TXA.

The surgeries were consistently performed by the same four senior surgeons. The NAC-carrying pedicles were sculpted according to different techniques depending on the preoperative conformation of the breast, including volume and ptosis. Hemostasis was achieved after reaching a mean arterial pressure of at least 75 mmHg corresponding to a normotensive range and checked bilaterally by the leading surgeon. In each breast, a 10-French drainage tube was placed in active aspiration. No compressive dressing or garments were positioned prior to hospital discharge. Postoperative pain was managed with the administration of paracetamol, ibuprofen, metamizole sodium, and eventually morphine. Ketorolac was not administered perioperatively.

During the postoperative phase, daily drainage volume and quality were collected at 6:30 AM by the ward nurses and checked by a resident doctor before 7:30 AM, as per standard postoperative protocol. Drains were removed when fluid production was less than or equal to 20 mL/24 h. After discharge, the outpatient visits were programmed on days 7–10 and a second visit between days 14 and 21. Data from further controls were not included because the aim of this study did not include analysis of later complications.

Thirteen variables were collected and analyzed for all patients to ensure the comparability between the two groups: age at time of surgery, BMI, history of type 2 diabetes mellitus (DM2), history of arterial hypertension, active smoking at time of surgery, hemoglobin, platelet count, international normalized ratio (INR), activated partial thromboplastin time (aPTT), use of antiaggregant and anticoagulant drugs prior to the surgery, as well as history of prior chemo- and radiotherapy were collected from the past medical history (Table 1).

The primary outcome of this study was the incidence of early bleeding requiring revisional surgery. The secondary outcomes were total drainage volume, time until drain removal, incidence of seroma formation, and wound dehiscence. Patient characteristics were categorized as either numeric or continuous variables. Numeric data with normal distributions were summarized using mean and standard deviation. Non-normally distributed numeric data or categorical data were described with frequencies and percentages. Categorical data were compared using chi-squared tests for large samples or Fisher’s exact test for small samples. For continuous data, parametric t-tests for normally distributed data and nonparametric Mann–Whitney U tests for non-normally distributed data were used. Results for continuous variables are presented as mean with corresponding 95% confidence intervals.

All statistical tests were performed two-sided, and *p*-value < 0.05 was considered statistically significant. Statistical analysis was performed using Stata version 17.0 software (StataCorp LP, College Station, TX, USA).

## 3. Results

A total of 157 patients underwent a total of 240 reduction mammaplasties. Of these, 31 breasts were excluded according to the previously defined criteria or due to incomplete data or patient dropout, resulting in a total of 209 breasts enrolled in the study, with 138 cases in the control group and 71 cases in the TXA group. Of the 138 cases in the control group, 88 were bilateral and 50 were unilateral cases. Of the 71 cases in the TXA group, 54 were bilateral and 17 were unilateral cases.

Descriptive and comparative statistical analysis of the two groups did not show statistically significant differences, and the groups were thus defined as comparable (Table 1).

No cases of bleeding requiring surgery were recorded in the TXA group (TXA group: 0% vs. control group: 2.3%). Although clinically relevant, this reduction did not reach statistical significance (*p* = 0.211).

Total drainage volume was significantly reduced by 22% in the TXA group compared to the control group (TXA group: 41.6 mL vs. control group: 53.8 mL; *p* = 0.012). A major reduction between 24 and 48 h after surgery was observed with a decrease of 23% (TXA group: 15.6 mL vs. control group: 20.5 mL; *p* = 0.013). In the first 24 h following surgery, a decrease of 17% in drainage volume was observed in the TXA group but did not reach statistical significance (TXA group: 21.6 mL vs. control group: 26.2 mL; *p* = 0.069).

Reduction of total drainage volume resulted in a significantly earlier drain removal by 0.6 days in patients administered TXA compared to the control group (TXA group: 1.6 days vs. control group: 2.2 days; *p* = 0.0001) (Table 2).

The incidence of wound dehiscence in the TXA group was slightly increased compared to the control group (5.6% vs. 3.6%) without reaching statistical significance. Seroma formation was marginally reduced in the TXA group without reaching statistical significance (0.0% vs. 0.7%).

Besides the common side effects of TXA (headache and nausea), no clinically observable thromboembolic events or other complications related to drug administration that necessitated interruption of TXA were observed throughout the entire follow-up period.

## 4. Discussion

Tranexamic acid (TXA), a common antifibrinolytic drug, is a synthetic analog of the amino acid lysine, which exhibits both antifibrinolytic and anti-inflammatory properties. These become significant in the post-traumatic acute phase of inflammation, such as seen after surgery, where different pathways contribute to fluid accumulation and edema in soft tissues [33,34].

This study showed that the applied 48 h TXA protocol significantly reduced postoperative drainage volume and, eventually, length of hospital stay in patients undergoing reduction mammaplasty. The most significant reduction in drainage volume occurred between 24 and 48 h following surgery, leading us to believe that the length of the protocol might also play a crucial role in reducing drainage volume, potentially through a prolonged inhibition of the inflammatory pathways reached by systemic administration of the drug.

This reduction in drainage volume translates into earlier removal of surgical drains and, thus, a shorter hospital stay. Accordingly, a significant reduction in the length of hospital stay by almost a day (0.6 days) was observed in the TXA group, with a mean of approximately 1.5 days. This results in a major impact, both on patient comfort and socioeconomic burden. Furthermore, earlier drain removal may reduce the elevated risk for complications that are related to remaining drains, such as pain, infection, and infection-related complications [35,36,37].

Regarding bleeding requiring revision surgery, no significant reduction was shown in the TXA group. We assume that this is due to two things. Firstly, the untreated group was associated with a rather low incidence of 2% of bleeding requiring surgical revision. Accordingly, a much larger patient group would have been needed to show the potential effect of TXA in postoperative bleeding prevention. Secondly, we believe that TXA is particularly efficient in avoiding bleeding that results from diffuse oozing, such as in patients in a hyperfibrinolytic state after trauma or cardiac surgery rather than from a single bleeding vessel [38,39].

Although no thromboembolic events were observed throughout the entire follow-up period, and previous studies have not shown an increase in the risk of thromboembolic events if administered in dosages comparable to that used in this study and respecting the given contraindications [31,32], further prospective trials are necessary to demonstrate the safety of TXA for this specific purpose.

As previously stated, multiple randomized studies have already established the importance of TXA administration to control excessive blood loss in the fields of traumatology, cardiac surgery, orthopedics, and gynecology [17,18,19,20,21,22]. In recent years, however, the plastic surgery community has shown an increasing interest in studying its administration in cases of soft tissue surgery, leading to an important increase in scientific evidence. Unfortunately, results were inconsistent regarding the reduction of postoperative bleeding and the formation of seromas. This may be due to the variability in the incidence of bleeding requiring revision surgery and due to some limitations in the studies, including the comparison of different administration protocols (i.e., dosage [40,41,42,43,44,45], systemic administration [40,42,43,44,45,46], topical application [41,46,47,48], or both [46,49,50]) or comparison of its effectiveness in different surgical procedures (i.e., mastectomy [40,43,44,45,47], reduction mammaplasty [41,46], lumpectomy [45], liposuction [49,50], and panniculectomy [48]).

In 2022, Calpin et al. published a meta-analysis, including a total of seven articles. The authors could thereby demonstrate that the incidence of postoperative hematoma was significantly lower in the groups that were administered TXA [31]. In 2023, Liechti et al. published another meta-analysis including five studies. Here, the authors were able to demonstrate a reduction in postoperative hematoma and seroma formation [32].

Although the results of both studies seem sound, we believe that the major limitation of these meta-analyses is based on the fact that many different types of breast procedures that yield different types of surgeries and eventually postoperative wounds have been compared, including reduction mammaplasty, breast-conserving surgery, modified radical mastectomy, and axillary lymph node dissection. Furthermore, it is striking to see a rather high rate of postoperative hematoma in both control and TXA groups of the publications discussed in the meta-analyses, compared to the one demonstrated by this study and those reported in other publications [7].

Conversely, only some months later, Weissler et al. published their results on the administration of TXA in reduction mammaplasty, concluding that TXA administration does not impact either postoperative bleeding or seroma formation but that it may reduce tissue inflammation. However, Weissler et al. compared different protocols of TXA administration (i.v. (1 g at the start of closure of the surgical wounds), topical (3 g in 75 mL of NaCl 0.9% directly onto the tissue and retrogradely through the drain following closure of the surgical wounds), and a combination of both) which were not implemented in a standardized manner but depended on the surgeons’ preference, thus making the study rather vulnerable to selection bias [46].

To overcome some of these limitations and to best reduce the possibility of confounding factors, only one protocol of combined i.v. and p.o. TXA administration was analyzed. This included, firstly, the type of breast surgery to be considered, which was always performed by the same surgeons (i.e., 4 senior surgeons), resulting in a similar type of postoperative wound and applied dressings. Secondly, the 48-h protocol of combined intravenous and per os administration of TXA was defined based on the literature available at that time [51], knowing that no consensus existed regarding dosage and duration of administration. TXA doses were respected to avoid possible neurological complications such as those demonstrated in other papers [52,53,54]. Nevertheless, this study could demonstrate that this 48-h protocol resulted in a significant reduction in postoperative drainage volume and, thus, earlier discharge from the hospital. The positive results presented in this study support the use of TXA in reduction mammaplasty and should prompt further investigation into different modes of administration, including whether a single topical administration leads to similar results or if a prolonged systemic protocol can reduce postoperative drainage volume even further. The combination of both topical and systemic application presents another promising regimen. Accordingly, one should also question the use of drains in reduction mammaplasty altogether, particularly if performed as a standalone procedure and not as an accompanying procedure to breast cancer surgery. In doing so, one could more easily offer the surgery as an outpatient procedure, which could facilitate the reimbursement policies in some countries like Switzerland or Germany for patients with symptomatic breast hypertrophy.

A major strength of this study is the collection and identification of various patient-related variables with the potential to influence the primary outcome of early postoperative bleeding requiring revision surgery. Analysis of these variables showed that there were no statistically significant differences between the two groups and demonstrated a relatively equal distribution and variance across the patient cohort.

This study has several limitations inherent to its retrospective design, including the potential for selection bias and confounding factors. To minimize these risks, all patients were drawn from the same cohort according to the previously mentioned criteria and received uniform treatment protocols. Furthermore, statistical adjustments were applied to account for potential confounders, though residual confounding cannot be entirely ruled out. Further prospective trials are needed for this specific purpose. In addition to these inherent limitations, this study presented a certain limitation regarding the type of drainage volume collection. Samples were evaluated every day with regard to volume and quality of output and recorded daily, identifying the fluid as haematic, sero-haematic, or serous for reference. However, no differentiation was made regarding the origin of the fluid output recorded. This poses a certain limitation as drainage volume is not purely haematic but also serous in nature, with the ratio of blood to serum differing each postoperative day and most bleeding occurring in the early postoperative days. This subjectivity leaves room for inaccurate blood loss quantification. However, this did not affect the registration of the primary outcome (postoperative bleeding) since this is defined as a major postoperative complication and necessitates further revision surgery. In a future study, laboratory analysis of the drainage fluid may be considered to precisely determine exact blood loss and better understand the role of TXA with regards to its antifibrinolytic or anti-inflammatory properties.

Furthermore, a subset of the patients was diagnosed with breast cancer and, therefore, were operated on one side for oncoplastic purposes, with the contralateral breast undergoing a reduction mammaplasty to achieve symmetry. Breasts that underwent reduction mammaplasties in these patients were still included and contributed to about 21% of both the treatment and control groups. It is unclear to what extent the pro-coagulative state of these patients may have affected their blood loss. However, it is important to note that previous research demonstrates no significant difference in postoperative bleeding between patients receiving TXA in the context of cancer surgery and those undergoing cosmetic surgery [31].

Lastly, it needs to be mentioned that four senior consultants performed all the surgeries. Accordingly, and despite the same surgical school, a certain bias may exist with regard to postoperative bleeding and oozing due to intraoperative variability. However, this limitation is mitigated by the strongly standardized techniques and postoperative procedures used in our department regarding reduction mammaplasty, hemostasis, surgical technique, and surgical time, thus rendering the different operations comparable nonetheless.

## 5. Conclusions

In conclusion, TXA is a well-tolerated drug with a good safety profile that significantly reduces post-surgical drainage volumes following reduction mammaplasty. This results in earlier drain removal and reduced length of hospital stay, and thus allows for a safer, faster, and more efficient recovery process postoperatively.

As seen from the results of other studies in centers with a higher rate of postoperative bleeding requiring revision surgery, the administration of TXA—be it topically, systemically, or both—can help control postoperative bleeding but should always be a supportive measure to the surgeon rather than presenting an alternative to well-executed surgery and hemostatic technique.

Given its safety profile and the important benefits for the patient, TXA should be widely used in plastic surgery, especially as trends in healthcare systems require physicians to promote outpatient procedures and quick postoperative recovery.

## Figures and Tables

**Table 1:** Patient Characteristics.

Variables	Control	TXA	*p*-Value
Age (years) ^a^	55.2 (15.7)	52.3 (16.5)	0.210 ^c^
BMI (kg/m^2^) ^a^	26.5 (5.8)	27.5 (5.6)	0.201 ^c^
History of DM2 ^b^	6 (4.3)	2 (2.8)	0.585 ^d^
History of arterial hypertension ^b^	27 (19.6)	12 (26.9)	0.640 ^d^
Active smoking ^b^	35 (25.5)	24 (33.8)	0.210 ^d^
Haemoglobin (mg/dL) ^a^	134.0 (11.4)	133.2 (15.2)	0.685 ^c^
Platelet count (mcL) ^a^	274.7 (69.3)	268.4 (60.9)	0.572 ^c^
INR ^a^	0.9 (0.1)	1 (0.1)	0.012 ^c^
aPTT ^a^	30.7 (4.5)	29.7 (3.7)	0.198 ^c^
Anti-aggregant drugs (suspended preoperatively) ^b^	10 (7.2)	5 (7)	0.957 ^d^
Anticoagulant drugs (suspended preoperatively) ^b^	4 (3)	0 (0)	0.268 ^d^
History of radiotherapy ^b^	7 (5.1)	1 (1.4)	0.191 ^d^
Prior neoadjuvant chemotherapy ^b^	14 (10.1)	9 (12.7)	0.580 ^d^

^a^ Mean (SD), ^b^ N (%), ^c^ *t*-test, ^d^ Chi^2^-test.

**Table 2:** Outcome data of primary and secondary endpoints.

Variables	Control	TXA	*p*-Value
Bleeding requiring surgical revision (%)	2.3	0.0	0.211 ^a^
Total drainage volume (mL)	53.8	41.6	0.012 ^b^
Drainage volume after 24 h (mL)	26.2	21.6	0.069 ^b^
Drainage volume between 24 and 48 h (mL)	20.5	15.6	0.013 ^b^
Length of hospital stay (days)	2.2	1.6	0.0001 ^b^
Seroma (%)	0.7	0.0	0.472 ^a^
Wound dehiscence (%)	3.6	5.6	0.498 ^a^

^a^ Chi^2^-test, ^b^ Mann–Whitney U-test.

## Data Availability

The data presented in this study are available on request from the corresponding author due to privacy reasons.

## References

[1] Blomqvist L., Eriksson A., Brandberg Y. (2000). Reduction Mammaplasty Provides Long-Term Improvement in Health Status and Quality of Life. Plast. Reconstr. Surg..

[2] Rogliani M., Gentile P., Labardi L., Donfrancesco A., Cervelli V. (2009). Improvement of physical and psychological symptoms after breast reduction. J. Plast. Reconstr. Aesthet. Surg..

[3] Saariniemi K.M.M., Keranen U.H., Salminen-Peltola P.K., Kuokkanen H.O.M. (2008). Reduction mammaplasty is effective treatment according to two quality of life instruments. A prospective randomised clinical trial. J. Plast. Reconstr. Aesthet. Surg..

[4] Gonzalez F., Walton R.L., Shafer B., Matory W.E., Borah G.L. (1993). Reduction mammaplasty improves symptoms of macromastia. Plast. Reconstr. Surg..

[5] Spector J.A., Karp N.S. (2007). Reduction mammaplasty: A significant improvement at any size. Plast. Reconstr. Surg..

[6] Daar D.A., Bekisz J.M., Chiodo M.V., DeMitchell-Rodriguez E.M., Saadeh P.B. (2021). Hematoma After Non-Oncologic Breast Procedures: A Comprehensive Review of the Evidence. Aesthetic Plast. Surg..

[7] Hall-Findlay E.J., Shestak K.C. (2015). Breast Reduction. Plast. Reconstr. Surg..

[8] Cunningham B.L., Gear A.J.L., Kerrigan C.L., Collins E.D. (2005). Analysis of breast reduction complications derived from the BRAVO study. Plast. Reconstr. Surg..

[9] Palve J., Kuuskeri M., Luukkaala T., Suorsa E. (2022). Predictive risk factors of complications in reduction mammoplasty—Analysis of three different pedicles. Gland. Surg..

[10] Rancati A., Irigo M., Angrigiani C. (2016). Management of the Ischemic Nipple-Areola Complex After Breast Reduction. Clin. Plast. Surg..

[11] Stanek K., Nussbaum L., Labow B.I., Chacko S., Ganske I.M., Ganor O., Vinson A., Greene A.K., Nuzzi L., Rogers-Vizena C.R. (2024). Understanding Hematoma Risk: Study of Patient and Perioperative Factors in a Large Cohort of Young Women Undergoing Reduction Mammaplasty. J. Am. Coll. Surg..

[12] Liu D., Wu M., Xu X., Luo L., Feng J., Ou Y., Zhang Y., Panayi A.C., Cui Y. (2023). Risk Factors and Complications in Reduction Mammaplasty: A Systematic Review and Meta-analysis. Aesthetic Plast. Surg..

[13] Anzarut A., Edwards D.C., Calder K., Guenther C.R., Tsuyuki R. (2008). Superior pedicle breast reduction techniques increase the risk of postoperative drainage. Ann. Plast. Surg..

[14] Manahan M.A., Buretta K.J., Chang D., Mithani S.K., Mallalieu J., Shermak M.A. (2015). An outcomes analysis of 2142 breast reduction procedures. Ann. Plast. Surg..

[15] Villani F., Caviggioli F., Banzatti B., Bandi V., Maione L. (2009). Correlation between complication rate and perioperative risk-factors in superior pedicle reduction mammaplasty: Our experience in 127 patients. Acta Chir. Plast..

[16] Ngan P.G., Iqbal H.J., Jayagopal S., Sillitoe A.T., Dhital S.K., Juma A. (2009). When to use drains in breast reduction surgery?. Ann. Plast. Surg..

[17] Roberts I., Shakur H., Coats T., Hunt B., Balogun E., Barnetson L., Cook L., Kawahara T., Perel P., Prieto-Merino D. (2013). The CRASH-2 trial: A randomised controlled trial and economic evaluation of the effects of tranexamic acid on death, vascular occlusive events and transfusion requirement in bleeding trauma patients. Health Technol. Assess..

[18] Dewan Y., Komolafe E.O., Mejía-Mantilla J.H., Perel P., Roberts I., Shakur H. (2012). CRASH-3—Tranexamic acid for the treatment of significant traumatic brain injury: Study protocol for an international randomized, double-blind, placebo-controlled trial. Trials.

[19] Roberts I., Perel P., Prieto-Merino D., Shakur H., Coats T., Hunt B.J., Lecky F., Brohi K., Willett K. (2012). Effect of tranexamic acid on mortality in patients with traumatic bleeding: Prespecified analysis of data from randomised controlled trial. BMJ.

[20] Ker K., Edwards P., Perel P., Shakur H., Roberts I. (2012). Effect of tranexamic acid on surgical bleeding: Systematic review and cumulative meta-analysis. BMJ.

[21] Habbab L.M., Semelhago L., Lamy A. (2020). Topical Use of Tranexamic Acid in Cardiac Surgery: A Meta-Analysis. Thorac. Cardiovasc. Surg..

[22] Huang F., Wu D., Ma G., Yin Z., Wang Q. (2014). The use of tranexamic acid to reduce blood loss and transfusion in major orthopedic surgery: A meta-analysis. J. Surg. Res..

[23] Okamoto S., Hijikata-Okunomiya A., Wanaka K., Okada Y., Okamoto U. (1997). Enzyme-Controlling Medicines: Introduction. Semin. Thromb. Hemost..

[24] Lee J.H. (2022). Effect of Topical Tranexamic Acid on Seroma Formation in a Rat Mastectomy Model. Aesthetic Plast. Surg..

[25] Jennewein C., Tran N., Paulus P., Ellinghaus P., Eble J.A., Zacharowski K. (2011). Novel Aspects of Fibrin(ogen) Fragments during Inflammation. Mol. Med..

[26] Walker P.F., Foster A.D., Rothberg P.A., Davis T.A., Bradley M.J. (2018). Tranexamic acid decreases rodent hemorrhagic shock-induced inflammation with mixed end-organ effects. PLoS ONE.

[27] Teng Y., Feng C., Liu Y., Jin H., Gao Y., Li T. (2018). Anti-inflammatory effect of tranexamic acid against trauma-hemorrhagic shock-induced acute lung injury in rats. Exp. Anim..

[28] Jimenez J.J., Iribarren J.L., Lorente L., Rodriguez J.M., Hernandez D., Nassar I., Perez R., Brouard M., Milena A., Martinez R. (2007). Tranexamic acid attenuates inflammatory response in cardiopulmonary bypass surgery through blockade of fibrinolysis: A case control study followed by a randomized double-blind controlled trial. Crit. Care.

[29] Lei Y., Xie J., Huang Q., Huang W., Pei F. (2020). Additional benefits of multiple-dose tranexamic acid to anti-fibrinolysis and anti-inflammation in total knee arthroplasty: A randomized controlled trial. Arch. Orthop. Trauma. Surg..

[30] Peng Z., Ban K., LeBlanc A., Kozar R.A. (2016). Intraluminal Tranexamic Acid Inhibits Intestinal Sheddases and Mitigates Gut and Lung Injury and Inflammation in a Rodent Model of Hemorrhagic Shock. J. Trauma. Acute Care Surg..

[31] Calpin G.G., McAnena P.F., Davey M.G., Calpin P., Kerin M.J., McInerney N., Walsh S.R., Lowery A.J. (2023). The role of tranexamic acid in reducing post-operative bleeding and seroma formation in breast surgery: A meta-analysis. Surgeon.

[32] Liechti R., Van De Wall B.J.M., Hug U., Fritsche E., Franchi A. (2023). Tranexamic Acid Use in Breast Surgery: A Systematic Review and Meta-Analysis. Plast. Reconstr. Surg..

[33] Cotran R.S., Robbins S.L., Kumar V., Abbas A.K., Aster J.C. (2021). Robbins & Cotran Pathologic Basis of Disease.

[34] compendium.ch. https://compendium.ch/product/1541204-tranexamic-orpha-inj-los-1000-mg-10ml/mpro.

[35] Xue D.Q., Qian C., Yang L., Wang X.F. (2012). Risk factors for surgical site infections after breast surgery: A systematic review and meta-analysis. Eur. J. Surg. Oncol..

[36] Murray J.D., Elwood E.T., Jones G.E., Barrick R., Feng J. (2009). Decreasing expander breast infection: A new drain care protocol. Can. J. Plast. Surg..

[37] Hanna K.R., Tilt A., Holland M., Colen D., Bowen B., Stovall M., Lee A., Wang J., Drake D., Lin K. (2016). Reducing Infectious Complications in Implant Based Breast Reconstruction: Impact of Early Expansion and Prolonged Drain Use. Ann. Plast. Surg..

[38] Pabinger I., Fries D., Schöchl H., Streif W., Toller W. (2017). Tranexamic acid for treatment and prophylaxis of bleeding and hyperfibrinolysis. Wien. Klin. Wochenschr..

[39] Fawzy H., Elmistekawy E., Bonneau D., Latter D., Errett L. (2009). Can local application of Tranexamic acid reduce post-coronary bypass surgery blood loss? A randomized controlled trial. J. Cardiothorac. Surg..

[40] Knight H., Banks J., Muchmore J., Ives C., Green M. (2019). Examining the use of intraoperative tranexamic acid in oncoplastic breast surgery. Breast J..

[41] Ausen K., Fossmark R., Spigset O., Pleym H. (2015). Randomized clinical trial of topical tranexamic acid after reduction mammoplasty. Br. J. Surg..

[42] Lardi A.M., Dreier K., Junge K., Farhadi J. (2018). The use of tranexamic acid in microsurgery—Is it safe?. Gland. Surg..

[43] Wolter A., Scholz T., Pluto N., Diedrichson J., Arens-Landwehr A., Liebau J. (2018). Subcutaneous mastectomy in female-to-male transsexuals: Optimizing perioperative and operative management in 8 years clinical experience. J. Plast. Reconstr. Aesthetic Surg..

[44] Lohani K.R., Kumar C., Kataria K., Srivastava A., Ranjan P., Dhar A. (2020). Role of tranexamic acid in axillary lymph node dissection in breast cancer patients. Breast J..

[45] Oertli D., Laffer U., Haberthuer F., Kreuter U., Harder F. (2005). Perioperative and postoperative tranexamic acid reduces the local wound complication rate after surgery for breast cancer. Br. J. Surg..

[46] Weissler J.M., Kuruoglu D., Antezana L., Curiel D., Kerivan L., Alsayed A., Banuelos J., Harless C.A., Sharaf B.A., Vijayasekaran A. (2022). Efficacy of Tranexamic Acid in Reducing Seroma and Hematoma Formation Following Reduction Mammaplasty. Aesthet. Surg. J..

[47] Ausen K., Hagen A.I., Østbyhaug H.S., Olafsson S., Kvalsund B.J., Spigset O., Pleym H. (2020). Topical moistening of mastectomy wounds with diluted tranexamic acid to reduce bleeding: Randomized clinical trial. BJS Open.

[48] Weissler J.M., Kuruoglu D., Salinas C., Tran N.V., Nguyen M.D.T., Martinez-Jorge J., Bite U., Harless C.A., Vijayasekaran A., Sharaf B. (2022). Defining the Role for Topically Administered Tranexamic Acid in Panniculectomy Surgery. Aesthet. Surg. J. Open Forum..

[49] El Minawi H.M., Kadry H.M., El-Essawy N.M., El Saadany Z.A., Nouh O.M. (2023). The effect of tranexamic acid on blood loss in liposuction: A randomized controlled study. Eur. J. Plast. Surg..

[50] Abboud N.M., Kapila A.K., Abboud S., Yaacoub E., Abboud M.H. (2021). The Combined Effect of Intravenous and Topical Tranexamic Acid in Liposuction: A Randomized Double-Blinded Controlled Trial. Aesthet. Surg. J. Open Forum.

[51] Rohrich R.J., Cho M.J. (2018). The Role of Tranexamic Acid in Plastic Surgery: Review and Technical Considerations. Plast. Reconstr. Surg..

[52] Pellegrini A., Giaretta D., Chemello R., Zanotto L., Testa G. (1982). Feline generalized epilepsy induced by tranexamic acid (AMCA). Epilepsia.

[53] Furtmüller R., Schlag M.G., Berger M., Hopf R., Huck S., Sieghart W., Redl H. (2002). Tranexamic acid, a widely used antifibrinolytic agent, causes convulsions by a gamma-aminobutyric acid(A) receptor antagonistic effect. J. Pharmacol. Exp. Ther..

[54] Schlag M.G., Hopf R., Zifko U., Redl H. (2002). Epileptic seizures following cortical application of fibrin sealants containing tranexamic acid in rats. Acta Neurochir..

